# Modified mallampati classification as a clinical predictor of peroral esophagogastroduodenoscopy tolerance

**DOI:** 10.1186/1471-230X-11-12

**Published:** 2011-02-15

**Authors:** Hsin-Hung Huang, Meei-Shyuan Lee, Yu-Lueng Shih, Heng-Cheng Chu, Tien-Yu Huang, Tsai-Yuan Hsieh

**Affiliations:** 1 Division of Gastroenterology, Department of Internal Medicine, Tri-Service General Hospital, National Defense Medical Center, Taiwan; 2 School of Public Health, National Defense Medical Center, Taiwan

## Abstract

**Background:**

Unsedated esophagogastroduodenoscopy (EGD) is simpler and safer than sedated EGD; however, approximately 40% of patients cannot tolerate it. Early identification of patients likely to poorly tolerate unsedated EGD is valuable for improving compliance. The modified Mallampati classification (MMC) has been used to evaluate difficult tracheal intubation and laryngoscope insertion. We tried to assess the efficacy of MMC to predict the tolerance of EGD in unsedated patients.

**Methods:**

Two hundred patients who underwent an unsedated diagnostic EGD were recruited. They were stratified according to the view of the oropharynx as either MMC class I + II (good view) or class III + IV (poor view). EGD tolerance was assessed in three ways: gag reflex by endoscopist assessment, patient satisfaction by interview, and the degree of change in vital signs.

**Results:**

MMC was significantly correlated to gag reflex (*P *< 0.001), patient satisfaction (*P *= 0.028), and a change of vital signs (*P *= 0.024). Patients in the poor view group had a 3.87-fold increased risk of gag reflex (*P *< 0.001), a 1.78-fold increased risk of unsatisfaction (*P *= 0.067), and a 1.96-fold increased risk of a change in vital signs (*P *= 0.025) compared to those in the good view group.

**Conclusions:**

MMC appears to be a clinically useful predictor of EGD tolerance. Patients with poor view of oropharynx by MMC criteria may be candidates for sedated or transnasal EGD.

## Background

Esophagogastroduodenoscopy (EGD) is a valuable screening, diagnostic, and therapeutic procedure for the upper gastrointestinal tract. However, nearly 40% of patients poorly tolerate unsedated EGD [1], and 10% of patients experience severe discomfort despite the use of an ultrathin endoscope [2,3]. Patient discomfort can interfere with the endoscopist's judgment and evoke cardiopulmonary complications, including cardiac arrhythmia, myocardial ischemia, aspiration, and hypoxemia [4-6]. Sedation can eliminate discomfort and increase patient compliance with EGD [7,8]. However, sedated EGD involves more time, monitoring, ancillary personnel, and has a higher cardiopulmonary risk than unsedated EGD [9], and is thus inappropriate for all patients. Hence, the early identification of patients potentially intolerant of unsedated EGD would improve clinical decision-making.

The Mallampati classification was first described in 1985 [10] and modified to include four categories in 1987 [11]. It is based on the poor visualization of the glottis when the tongue base is disproportionately large and predicts difficult tracheal intubation and laryngoscope insertion. The EGD involves the same route as tracheal intubation and laryngoscopy, and is associated with the same discomforts, such as oropharyngeal irritation, retching, and gag reflex. Therefore, it is reasonable to link the modified Mallampati classification (MMC) and peroral EGD tolerance. To test the usefulness of MMC in clinical practice for routine diagnostic EGD, we designed the present study to assess the tongue base size and the view of the oropharynx by using the MMC to predict EGD tolerance in unsedated patients.

## Methods

### Patients

From October 2008 to May 2009, two hundred eligible patients (97 men and 103 women; age 18 - 86 years) who were not mentally incompetent, did not use sedatives or beta-blockers, had not had an emergency endoscopic procedure, or a history of oropharyngeal surgery agreed to participate in the present study. For sample size calculation, the minimally clinically significant difference in rates of gag reflex between two MMC groups was considered to be 15%. Thus 60 patients were required to give a 90% power to detect a 15% difference at the 5% significance level for a two-sided test. The study protocol was approved by the Institutional Review Board of Tri-Service General Hospital, Taiwan.

### Data collection

Before unsedated diagnostic EGD, a written informed consent and a detailed medical history from patients were obtained by an endoscopist followed by a face-to-face interview and MMC status evaluation by two trained research nurses. Information collected included age, gender, body mass index (BMI), education (up to high school; college and above), smoking status, pre-EGD anxiety, previous EGD experience and satisfaction. Both patients and endoscopists were blinded to MMC status.

All patients received 4 puffs of topical pharyngeal spray containing 10% lidocaine hydrochloride (Xylocaine Viscous, Astra, Sweden) 5 and 2 minutes before EGD for a total dosage of 80 mg. Anti-peristaltic agents or glucagon were not used before EGD. All EGD procedures used Olympus GIF-Q260, XQ260, and XQ240 videoendoscopes with 9.0 - 9.2 mm outer diameter of distal tip (Olympus Optical Co. Ltd., Tokyo, Japan) in 109, 82, 9 patients respectively, and were performed by attending endoscopists. Throughout the procedure, noninvasive mean blood pressure (MBP), pulse rate (PR), and peripheral oxygen saturation (SaO_2_) were monitored with an automated system (Philips, MP20 Junior and C1, Germany).

After EGD, endoscopists immediately assessed the presentation of gag reflex and the main diagnosis of EGD. Patients completed questionnaires prior to leaving the endoscopy center.

### Measurements

#### Modified Mallampati classification (MMC)

**Figure 1:**
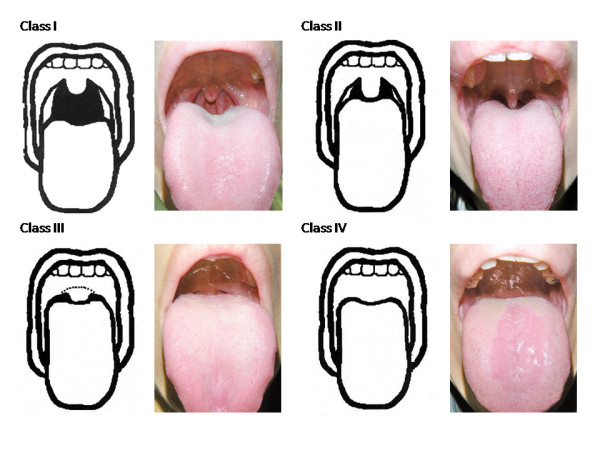
**Modified Mallampati classification of oropharyngeal visualization**. Class I: Class II: Class III: Class IV:

MMC assessment was performed with the patient sitting upright with his or her mouth maximally opened and tongue protruded without phonation by two trained research nurses. The participants were assigned to four classes (Figure 1): [11]

Class I: Soft palate, fauces, pillars, and uvula are visible.

Class II: Soft palate, fauces, and uvula are visible.

Class III: Soft palate and base of uvula are visible.

Class IV: Soft palate is not visible at all.

Class I and II were defined as "good view" and III and IV as "poor view" in the present study.

Reliability of MMC classification between observers was evaluated by agreement for the independent classifications of 80 subjects from two observers. Kappa values were 0.731 and 0.974 for four (I, II, III, IV) and two (good view and poor view) MMC categories, respectively.

#### EGD tolerance assessment

EGD tolerance was evaluated on the basis of *endoscopist assessment*, *patient satisfaction*, and *a change in vital signs*.

#### Endoscopist assessment

When the endoscopist felt a difficult intubation with interruption by obvious retching or vomiting, the assessment for gag reflex was recorded as "present". The main diagnosis were normal, gastroesophageal reflux disease (GERD), peptic ulcer disease (PUD), and both GERD and PUD.

#### Patient satisfaction

Information on self-assessed satisfaction was based on the following questions: "Were you satisfied with the unsedated EGD?" The response was "satisfactory" or "unsatisfactory"; "Would you be willing to undergo unsedated EGD again?" The response was "yes" or "no".

#### A change of vital signs

We recorded MBP, PR, and SaO_2 _before EGD and when the endoscope was in the middle third of the esophagus (about 25 cm from the incisors). A change in vital signs was defined as an increase in MPB or PR by 20% or more, or a decrease in SaO_2 _to 90% or less between the two recordings.

### Statistical analysis

The data are presented as mean ± standard deviation (SD) or percentage for continuous variables and categorical variables, respectively. The demographic and clinical characteristics of the subjects between groups were assessed by Student's t tests (continuous variables) and chi-square tests (categorical variables). Logistic regression analyses adjusting for possible covariates were used to evaluate the relative risk of MMC for the three methods to assess EGD tolerance. All statistical analyses were two-tailed. Statistical significance was accepted at 5% probability level. Analyses were done with the SPSS statistical analysis program, version 15.0.

## Results

The 200 patients had a mean age of 49.2 years and BMI of 23.6 kg/m^2^. In this study, 43.5% patients were college graduates, 72.5% were non-smokers, 73.0% had no pre-EGD anxiety, 64.5% had previously undergone EGD, and 49.6% were satisfied with a previous experience of EGD. After the EGD, 52.5% patients felt satisfied, 75.5% were willing to undergo repeat unsedated EGD, 72.5% had no gag reflex, 52.5% experienced less than 20% MBP elevation from baseline, 50.0% experienced less than 20% PR elevation from baseline, and 100% maintained an SaO_2 _level over 90%. GERD was diagnosed in 67.5% patients, PUD in 13.5%, and both GERD and PUD in 8.5%, while 10.5% patients were normal (data not shown).

More than 60% (123 out of 200) of patients were in the poor view group (class III + IV). Patients in the poor view group had a significantly higher mean BMI than those in the good view group (class I + II) (24.1 vs. 22.7 kg/m^2^; *P *= 0.01) (Table 1).

**Table 1:** Comparison of clinical and demographic characteristics by modified Mallampati classification (MMC)

	MMC class		
		
Descriptor	Good view (n = 77)	Poor view (n = 123)	*P *value
Male gender	46.8	49.6	0.696
Mean age (years), mean ± SD	50.7 ± 17.5	48.2 ± 16.0	0.301
BMI (kg/m^2^), mean ± SD	22.7 ± 3.4	24.1 ± 3.9	0.010
Up to high school education	58.4	55.3	0.661
Smoker	20.8	31.7	0.092
Previous EGD experience			0.179
No	31.1	38.2	
Good	40.3	27.6	
Poor	28.6	34.1	
Pre-EGD anxiety	26.0	28.5	0.702
EGD diagnosis			0.155
Normal	16.9	7.3	
GERD	66.2	68.3	
PUD	9.1	13.8	
Both (GERD + PUD)	7.8	10.6	
Types of endoscope			0.239
Q260 (9.2 mm)	59.7	51.2	
XQ260 + XQ240 (9.0 mm)	40.3	48.8	

Values reflect % unless otherwise noted. Good view: I + II; Poor view: III + IV; BMI, body mass index; EGD, esophagogastroduodenoscopy; GERD, gastroesophageal reflux disease; PUD, peptic ulcer disease.

The clinical characteristics for different gag reflex status, judged by the endoscopist, are shown in Table 2. Fifty-five (27.5%) patients presented gag reflex during EGD. The proportion of patients with a poor view of the oropharynx was significantly higher in the gag reflex present group than the gag reflex absent group (81.8% vs. 53.8%, *P *< 0.001). Moreover, though not significant, there were more males (58.2% vs. 44.8%), educated (54.5% vs. 39.3%) and smokers (36.4% vs. 24.1%) in the gag reflex present group.

**Table 2:** Comparison of clinical and demographic characteristics by presence of gag reflex

	Endoscopist assessment for gag reflex		
		
Descriptor	Absent (n = 145)	Present (n = 55)	*P *value
MMC class, poor view	53.8	81.8	<0.001
Male gender	44.8	58.2	0.092
Mean age (years), mean ± SD	50.2 ± 16.9	46.5 ± 15.5	0.158
BMI (kg/m2), mean ± SD	23.6 ± 3.7	23.4 ± 4.1	0.689
Up to high school education	60.7	45.5	0.052
Smoker	24.1	36.4	0.084
Previous EGD experience			0.324
No	32.4	43.6	
Good	34.5	27.3	
Poor	33.1	29.1	
Pre-EGD anxiety	27.6	27.3	0.965
EGD diagnosis			0.192
Normal	13.1	5.5	
GERD	68.3	65.5	
PUD	9.7	18.2	
Both (GERD + PUD)	9.0	10.9	
Types of endoscope			0.520
Q260 (9.2 mm)	53.1	58.2	
XQ260 + XQ240 (9.0 mm)	46.9	41.8	

Values reflect % unless otherwise noted. MMC, modified Mallampati classification; Good view: I + II; BMI, body mass index; EGD, esophagogastroduodenoscopy; GERD, gastroesophageal reflux disease; PUD, peptic ulcer disease.

More patients with unsatisfactory EGD were found in the poor view group (45.7% vs. 30.5%, *P *= 0.028), were younger (*P *= 0.012), had no or poor previous EGD experience (*P *= 0.004), and had pre-EGD anxiety (38.9% vs. 17.1%, *P *= 0.001) (Table 3). The proportion of patients who had a change in vital signs during EGD was significantly higher in the poor view group than in the good view group (67.5% vs. 51.4%, *P *= 0.024) (Table 4).

**Table 3:** Comparison of clinical and demographic characteristics by patient satisfaction status

	Patient satisfaction		
		
Descriptor	Satisfactory (n = 105)	Unsatisfactory (n = 95)	*P *value
MMC class, poor view	54.3	69.5	0.028
Male gender	51.4	45.3	0.384
Mean age (years), mean ± SD	51.9 ± 16.5	46.1 ± 16.2	0.012
BMI (kg/m^2^), mean ± SD	23.8 ± 3.7	23.4 ± 3.9	0.447
Up to high school education	62.9	49.5	0.057
Smoker	72.4	72.6	0.968
Previous EGD experience			0.004
No	30.5	41.1	
Good	42.9	21.1	
Poor	26.7	37.9	
Pre-EGD anxiety	17.1	38.9	0.001
EGD diagnosis			0.850
Normal	11.4	10.5	
GERD	69.5	65.3	
PUD	10.5	13.7	
Both (GERD + PUD)	8.6	10.5	
Types of endoscope			0.949
Q260 (9.2 mm)	54.3	54.7	
XQ260 + XQ240 (9.0 mm)	45.7	45.3	

Values reflect % unless otherwise noted. MMC, modified Mallampati classification; Good view: I + II; BMI, body mass index; EGD, esophagogastroduodenoscopy; GERD, gastroesophageal reflux disease; PUD, peptic ulcer disease.

**Table 4:** Association between patients' clinical and demographic characteristics and a change in vital signs

	A change in vital signs (≥20%)		
		
Descriptor	No (n = 74)	Yes (n = 126)	*P *value
MMC class, poor view	51.4	67.5	0.024
Male gender	50.0	47.6	0.745
Mean age (years), mean ± SD	52.2 ± 17.2	47.4 ± 16.0	0.050
BMI (kg/m^2^), mean ± SD	23.8 ± 4.1	23.5 ± 3.6	0.578
Up to high school education	58.1	55.6	0.725
Smoker	77.0	69.8	0.272
Previous EGD experience			0.469
No	36.5	34.9	
Good	36.5	30.2	
Poor	27.0	34.9	
Pre-EGD anxiety	31.1	25.4	0.385
EGD diagnosis			0.203
Normal	13.5	9.5	
GERD	64.9	69.0	
PUD	16.2	9.5	
Both (GERD + PUD)	5.4	11.9	
Types of endoscope			0.623
Q260 (9.2 mm)	56.8	53.2	
XQ260 + XQ240 (9.0 mm)	43.2	46.8	

Values reflect % unless otherwise noted. MMC, modified Mallampati classification; Good view: I + II; BMI, body mass index; EGD, esophagogastroduodenoscopy; GERD, gastroesophageal reflux disease; PUD, peptic ulcer disease.

The sensitivities and specificities of MMC in predicting the gag reflex, patient satisfaction and a change in vital signs were 81.8%, 69.5%, 67.5% and 46.2%, 45.7%, 48.6%, respectively (data not shown). After adjusting for potential covariates, by comparison with patients in the good view group, those in the poor view group had a 3.87-fold relative risk (95% confidence interval (CI): 1.81 - 8.26, *P *< 0.001) of experiencing a gag reflex; a 1.78-fold increased risk of unsatisfaction (95% CI: 0.96 - 3.29, *P *= 0.067); a 1.96-fold greater risk of experiencing a change in vital signs (95% CI: 1.09 - 3.54, *P *= 0.025) (Table 5).

**Table 5:** Odds ratio (OR) and 95% confidence interval (CI) for esophagogastroduodenoscopy tolerance by modified Mallampati classification (MMC)

	MMC status, OR (95% CI)		
Outcome			*P *value
	Good view	Poor view	
Endoscopist assessment for gag reflex (present vs. absent)	1	3.87 (1.81 - 8.26)	<0.001
Patient satisfaction (unsatisfactory vs. satisfactory)	1	1.78 (0.96 - 3.29)†	0.067
A change of vital signs (yes vs. no)	1	1.96 (1.09 - 3.54)	0.025

†Adjusting for age, previous EGD experience, and pre-EGD anxiety. Good view: I + II; Poor view: III + IV

## Discussion

Factors associated with poor EGD tolerance include young age [1,2,12-14], being female [13,15,16], a non-smoker [17], having low education [13,15], a poor previous EGD experience [15], and high anxiety prior to EGD [12,13,15,18,19]. To our knowledge, this report is the first demonstration that MMC is significantly associated with EGD tolerance as defined by endoscopist assessment, patient satisfaction, or a change in vital signs. MMC class III + IV can predict patients who are likely to suffer physical reactions (gag reflex, hypertension, and tachycardia) during EGD. This simple, fast and noninvasive method should allow the selection of patients who require sedated EGD.

EGD tolerance is a complex concept that encompasses a broad range of specific symptoms and expectations, and there is no current consensus on its definition. In our study, EGD tolerance consisted of three indicators which reflect assessments by the operator and receiver; objective reactions and subjective feelings; physical and mental effects. The willingness to repeat unsedated EGD is mainly depended on the need of therapy, doctor-patient relationships (75.5% were willing to repeat unsedated EGD but only 52.5% patients felt satisfied after the EGD in our study), or other available methods, such as sedated and transnasal EGD, which are limited by hospital facility, underlying conditions, and expenses. Thus, EGD tolerance did not include this item. A difficult intubation with interruption by gag reflex may result in violent peristalsis with poor images, the missing of minor lesions, gastroduodenal spasm with easy trauma by endoscope. Overall, endoscopist judgement and EGD quality are likely to be compromised. Endoscopist assessment may be the most important indicator of EGD tolerance for the clinical physician. Subjective predictors such as pre-EGD anxiety [12,13,15,18,19] and previous EGD experience [15] may be influenced by procedure time, volume of insufflated air into the stomach, frequent pushing and pulling of the endoscope, and the doctor-patient relationship. Patient satisfaction partially depended on the recall from pre-EGD anxiety and previous EGD experience and cannot, therefore, be an independent indicator of EGD tolerance. Our data show that MMC is significantly predictive of endoscopist assessment of gag reflex and of a change in vital signs, but not of patient satisfaction.

The tongue is the largest single structure in the oral cavity, and there is no practical bed-side method to measure its size objectively. A disproportionately large base of the tongue may mask the fauces and posterior part of the soft palate, overshadow the larynx, and result in loss of direction and a larger contact surface area for the EGD. Transnasal EGD is not passed through the oral cavity and greatly decrease the oropharyngeal irritation, such as choking, gag reflex, and sympathetic nervous activity [20,21]. Therefore, MMC can readily assess the view of the oropharyngeal space and accurately select candidates for the transnasal EGD.

We have compared the patient profile and diagnosis by status favorability within each of the three EGD tolerance measures, which has not been done collectively in previous studies. Both pre-EGD anxiety and previous EGD experience were only associated with patient satisfaction. Pre-EGD anxiety was related to previous EGD experience. In the present study, only 9.2% of patients with a good previous EGD experience suffered pre-EGD anxiety, which was less than those with a poor (40.6%) or no past experience (32.4%) (data not shown). Thus, an earlier positive EGD experience helps provide for low pre-EGD anxiety and improve satisfaction in a future EGD. Same as the previous study [22], our older patients have less oropharyngeal sensitivity and well tolerate to EGD procedure. The role of gender in EGD tolerance has been controversial [8,16]. Females report less satisfaction and males more frequently experience a gag reflex in our study. Contrary to previous studies [13,15], we found that patients with a higher education level had less satisfaction and more instances of gag reflex than those with a lower education level. This finding may be attributable to younger age (41.7 vs. 54.9 years) and to a predominance of males (59.8% vs. 39.8%). We, unlike other studies, did not find association between EGD tolerance and any of BMI, smoking status, types of endoscope, or EGD diagnosis. There are no studies discussing the association between the depth of mucosal injury and the EGD tolerance before. We inferred that superficial mucosal injury like atrophic gastritis or Helicobacter pylori related gastritis less affects the EGD tolerance as well as PUD with deeper mucosal injury. However, it is needed to be proved by further prospective studies in the future.

Ways of managing our findings might emerge from procedural and technical advances. Patients with the poor view of the oropharynx regardless of anxiety traits and EGD experiences, might be candidates for more widely acceptable methods for sedated EGD. Patients in whom the view is poor and unwilling or unsuitable for current methods of sedation, could have transnasal EGD with an ultrathin endoscope, more topical pharyngeal spray, or an improved view of the oropharynx by phonation or head-neck posture. There are several limitations in this study. First, our findings may not applicable to morbidly obese patients who tend to be in MMC class III + IV. These patients are likely to have comorbidities such as cardiovascular diseases, the obesity hypoventilation syndrome, and obstructive sleep apnea [23,24], and may be more safely examined by unsedated transnasal EGD than having sedated EGD [25]. Second, our study investigated diagnostic EGD without interventional procedures. Intravenous sedation is standard practice and an even better option in the performance of therapeutic EGD [26]. Finally, like a previous report of tracheal intubation and laryngoscopy [27], we observed that MMC has good sensitivity (0.68 - 0.82), but poor discriminative power in predicting EGD tolerance. Combination with other predictors may add further diagnostic value to the use of MMC.

## Conclusions

EGD is an inevitably unpleasant procedure but EGD tolerance is different for each patient. In conclusion, we have demonstrated that MMC is a clinically useful tool in the prediction of EGD tolerance in unsedated patients. Patients with a poor view of the oropharynx need consideration for sedated or transnasal EGD.

## Competing interests

The authors declare that they have no competing interests.

## Authors' contributions

HHH conceived of the study, involved in its design and coordination and drafted the manuscript. YLS, HCC, and TYH participated in the sequence alignment and helped to collect data. MShL participated in its design and performed the statistical analysis. TYH revised it critically for important intellectual content and helped to draft the manuscript. All authors read and approved the final manuscript.

## Additional files

Letter of approval by Institutional Review Board of Tri-Service General Hospital

## Pre-publication history

The pre-publication history for this paper can be accessed here:

http://www.biomedcentral.com/1471-230X/11/12/prepub

## References

[B1] Abraham N Barkun A Larocque M Fallone C Mayrand S Baffis V Cohen A Daly D Daoud H Joseph L Predicting which patients can undergo upper endoscopy comfortably without conscious sedation Gastrointest Endosc 2002 56 180 189 10.1016/S0016-5107(02)70175-2 12145594

[B2] Zaman A Hapke R Sahagun G Katon RM Unsedated peroral endoscopy with a video ultrathin endoscope: patient acceptance, tolerance, and diagnostic accuracy Am J Gastroenterol 1998 93 1260 1263 10.1111/j.1572-0241.1998.00406.x 9707048

[B3] Mulcahy HE Kelly P Banks MR Connor P Patchet SE Farthing MJ Fairclough PD Kumar PJ Factors associated with tolerance to, and discomfort with, unsedated diagnostic gastroscopy Scand J Gastroenterol 2001 36 1352 1357 10.1080/003655201317097245 11761029

[B4] Waring JP Baron TH Hirota WK Goldstein JL Jacobson BC Leighton JA Mallery JS Faigel DO Guidelines for conscious sedation and monitoring during gastrointestinal endoscopy Gastrointest Endosc 2003 58 317 322 10.1067/S0016-5107(03)00001-4 14528201

[B5] Levy N Abinader E Continuous electrocardiographic monitoring with Holter electrocardiocorder throughout all stages of gastroscopy Am J Dig Dis 1977 22 1091 1096 10.1007/BF01072863 930908

[B6] Quine MA Bell GD McCloy RF Charlton JE Devlin HB Hopkins A Prospective audit of upper gastrointestinal endoscopy in two regions of England: safety, staffing, and sedation methods Gut 1995 36 462 467 10.1136/gut.36.3.462 7698711 PMC1382467

[B7] Fisher NC Bailey S Gibson JA A prospective, randomized controlled trial of sedation vs. no sedation in outpatient diagnostic upper gastrointestinal endoscopy Endoscopy 1998 30 21 24 10.1055/s-2007-993723 9548039

[B8] Froehlich F Schwizer W Thorens J Kohler M Gonvers JJ Fried M Conscious sedation for gastroscopy: patient tolerance and cardiorespiratory parameters Gastroenterology 1995 108 697 704 10.1016/0016-5085(95)90441-7 7875472

[B9] Lazzaroni M Bianchi PG Preparation, premedication, and surveillance Endoscopy 2005 37 101 109 10.1055/s-2004-826149 15692924

[B10] Mallampati SR Gatt SP Gugino LD Desai SP Waraksa B Freiberger D Liu PL A clinical sign to predict difficult tracheal intubation: a prospective study Can Anaesth Soc J 1985 32 429 434 10.1007/BF03011357 4027773

[B11] Samsoon GL Young JR Difficult tracheal intubation: a retrospective study Anaesthesia 1987 42 487 490 10.1111/j.1365-2044.1987.tb04039.x 3592174

[B12] Tan CC Freeman JG Throat spray for upper gastrointestinal endoscopy is quite acceptable to patients Endoscopy 1996 28 277 282 10.1055/s-2007-1005453 8781790

[B13] Mahajan RJ Johnson JC Marshall JB Predictors of patient cooperation during gastrointestinal endoscopy J Clin Gastroenterol 1997 24 220 223 10.1097/00004836-199706000-00007 9252844

[B14] Farhadi A Fields JZ Hoseini SH The assessment of esophagogastroduodenoscopy tolerance a prospective study of 300 cases Diagn Ther Endosc 2001 7 141 147 10.1155/DTE.7.141 18493558 PMC2362842

[B15] Campo R Brullet E Montserrat A Calvet X Moix J Rue M Roque M Donoso L Bordas JM Identification of factors that influence tolerance of upper gastrointestinal endoscopy Eur J Gastroenterol Hepatol 1999 11 201 204 10.1097/00042737-199902000-00023 10102233

[B16] Dumortier J Napoleon B Hedelius F Pellissier PE Leprince E Pujol B Ponchon T Unsedated transnasal EGD in daily practice: results with 1100 consecutive patients Gastrointest Endosc 2003 57 198 204 10.1067/mge.2003.59 12556784

[B17] Gelly AL Farley A Boyer J Asselin M Spenard J Influence of sex, age and smoking status on patient comfort during gastroscopy with pharyngeal anesthesia by a new benzocaine-tetracaine preparation Can J Gastroenterol 1998 12 431 433 9784899 10.1155/1998/395953

[B18] Soma Y Saito H Kishibe T Takahashi T Tanaka H Munakata A Evaluation of topical pharyngeal anesthesia for upper endoscopy including factors associated with patient tolerance Gastrointest Endosc 2001 53 14 18 10.1067/mge.2001.111773 11154482

[B19] Faulx AL Catanzaro A Zyzanski S Cooper GS Pfau PR Isenberg G Wong RC Sivak MV Jr Chak A Patient tolerance and acceptance of unsedated ultrathin esophagoscopy Gastrointest Endosc 2002 55 620 623 10.1067/mge.2002.123274 11979240

[B20] Dean Robert Kulwinder Dua Massey Benson Berger William Walter J Hogan Reza Shaker A comparative study of unsedated transnasal esophagogastroduodenoscopy and conventional EGD Gastrointest Endosc 1996 44 422 424 10.1016/S0016-5107(96)70092-5 8905361

[B21] Yagi J Adachi K Arima N Tanaka S Ose T Azumi T Sasaki H Sato M Kinoshita Y A prospective randomized comparative study on the safety and tolerability of transnasal esophagogastroduodenoscopy Endoscopy 2005 37 1226 1231 10.1055/s-2005-921037 16329022

[B22] Davies AE Kidd D Stone SP MacMahon J Pharyngeal sensation and gag reflex in healthy subjects Lancet 1995 345 487 488 10.1016/S0140-6736(95)90584-7 7861875

[B23] Yusuf S Hawken S Ounpuu S Dans T Avezum A Lanas F McQueen M Budaj A Pais P Varigos J Lisheng L Effect of potentially modifiable risk factors associated with myocardial infarction in 52 countries (the INTERHEART study): case-control study Lancet 2004 364 937 952 10.1016/S0140-6736(04)17018-9 15364185

[B24] Poulain M Doucet M Major GC Drapeau V Series F Boulet LP Tremblay A Maltais F The effect of obesity on chronic respiratory diseases: pathophysiology and therapeutic strategies CMAJ 2006 174 1293 1299 16636330 10.1503/cmaj.051299 PMC1435949

[B25] Adams JP Murphy PG Obesity in anaesthesia and intensive care Br J Anaesth 2000 85 91 108 10.1093/bja/85.1.91 10927998

[B26] Faigel DO Baron TH Goldstein JL Hirota WK Jacobson BC Johanson JF Leighton JA Mallery JS Peterson KA Waring JP Fanelli RD Wheeler-Harbaugh J Guidelines for the use of deep sedation and anesthesia for GI endoscopy Gastrointest Endosc 2002 56 613 617 10.1016/S0016-5107(02)70104-1 12397263

[B27] Restelli L Moretti MP Todaro C Banfi L The Mallampati's scale: a study of reliability in clinical practice Minerva Anestesiol 1993 59 261 265 8355867

